# Investigation of optimum *N*-terminal probrain natriuretic peptide level in patients on maintained hemodialysis

**DOI:** 10.1080/0886022X.2017.1287732

**Published:** 2017-02-16

**Authors:** Lan Chen, Ying-Ying Chen, Yi-Sheng Ling, Chun-Hua Lin, Jin-Xuan He, Tian-Jun Guan

**Affiliations:** Department of Nephrology, Zhongshan Hospital, Xiamen University, Xiamen, Fujian, China

**Keywords:** Blood pressure, dry weight, hemodialysis, hemoglobin, *N*-terminal probrain natriuretic peptide

## Abstract

**Background:** Serum *N*-terminal probrain natriuretic peptide (NT-proBNP) level is known to be strongly associated with fluid overload, and serves as a guide for fluid management in patients on hemodialysis (HD). This study aimed at investigating the relationship between NT-proBNP level and blood pressure (BP), ultrafiltration/dry weight ratio as well as hemoglobin, and to explore the optimal cutoff point of NT-proBNP level in Chinese patients on HD.

**Methods:** A total of 306 patients on maintained HD for stage 5 chronic kidney disease (CKD) were included in this prospective study. Their average ultrafiltration/dry weight ratio and BP before dialysis were recorded. The serum NT-proBNP, hemoglobin, serum calcium, and phosphorus were detected. The cutoff value for NT-proBNP level was calculated using receiver operating characteristic (ROC) analysis.

**Results:** The high NT-proBNP level was associated with high BP and ultrafiltration/dry weight ratio, and low hemoglobin level. The optimal cutoff point of NT-proBNP level for patients on maintained HD was 5666 pg/mL, with a sensitivity of 78.5%, specificity of 43.9%, and area under the curve (AUC) of 0.703 (<0.001).

**Conclusions:** NT-proBNP level ≤5666 pg/mL was recommended to achieve the target BP, hemoglobin level, and ultrafiltration/dry weight ratio in patients on maintained HD with an ejection fraction (EF) >50%.

## Introduction

Chronic kidney disease (CKD) has become a major health concern, with a high prevalence worldwide. It has been estimated that nearly 1 in 10 people have some degree of kidney dysfunction in China, affecting almost 150 million Chinese individuals.^1^ Fluid overload has recently been shown to increase adverse outcomes in patients on hemodialysis (HD).^2–5^ Patients on maintained HD are often in a condition of extreme volume shifts, and adverse events such as interdialytic weight gain, rapid fluid removal during HD, and chronic fluid overload increase the strain or instability of the cardiovascular system.^6^ Therefore, accurate assessment of volume status is an integral component to achieve fluid balance in HD treatment.^7^ In addition, chronic volume expansion results in hypertension, left ventricular hypertrophy, and heart failure in patients on HD.^8^^,^^9^ The recently study of Dialysis Outcome and Practice Pattern Study (DOPPS) has shown the median ratios of patients with coronary artery disease and congestive heart failure in maintained HD patients were 34% and 24%, respectively.^10^ On the contrary, volume insufficiency may predispose the patient to intradialytic hypotension, muscle spasm, arrhythmias, stethalgia, and discomfort after treatment.^8^ Therefore, control of adequate extracellular fluid volume is important for regulation of blood pressure (BP) and prevention of cardiovascular complications in the Chinese population.^11^

Brain natriuretic peptide (BNP) is a kind of cardiac peptide secreted by ventricles of the myocardium in response to excessive myocardial stretching and abnormal wall tension. Elevated BNP levels have been shown to contribute to increased risk of mortality in patients with end-stage renal disease.^12^ In patients on maintained HD, BNP levels are commonly used to predict fluid overload.^13^^,^^14^*N*-terminal probrain natriuretic peptide (NT-proBNP) is secreted in equivalent proportion to BNP. Similar to BNP, NT-proBNP level is known to be strongly associated with fluid overload,^12^^,^^15^^,^^16^ and thus serves as a guide for fluid management in patients on HD.^17^ NT-pro BNP level might also be a very helpful biomarker in screening for left-ventricular diastolic dysfunction and ejection fraction (EF) and determining the need for echocardiography or a sophisticated cardiac study in HD patients.^18^ Several clinical laboratories in Chinese hospitals test NT-proBNP instead of BNP to detect early-stage left ventricular dysfunction, probably owing to the longer half-life and higher sensitivity of NT-proBNP.^19^ In addition, since BNP is metabolized by the liver whereas NT-proBNP is metabolized by the kidney,^20^ NT-proBNP level seems to be higher in patients with renal dysfunction, especially in patients on maintained HD, than in the healthy population. Currently, there are still no formally recommended NT-proBNP levels for patients on HD.

Nearly 90% of patients with advanced CKD (defined as stage 4 and 5 CKD) have anemia.^21^ The severity of anemia has been well correlated with the decline in glomerular filtration rate (GFR).^22^ Early management of anemia may decrease the mortality as well as improve the quality of life of patients with CKD. Elevated NT-pro-BNP level has been found in patients with severe anemia and longer total duration of HD.^23^ NT-proBNP was highly significant correlation with hemoglobin and significant correlation with estimated glomerular filtration rate in CKD patients.^24^ However, the mechanism is unknown.

In the present study, we investigated the relationship between NT-proBNP level and BP, ultrafiltration/dry weight ratio as well as hemoglobin in a single-center population of patients on HD, and explored the optimal cutoff point of NT-proBNP level in Chinese patients on HD.

## Methods and materials

### Study population

**Figure 1.**
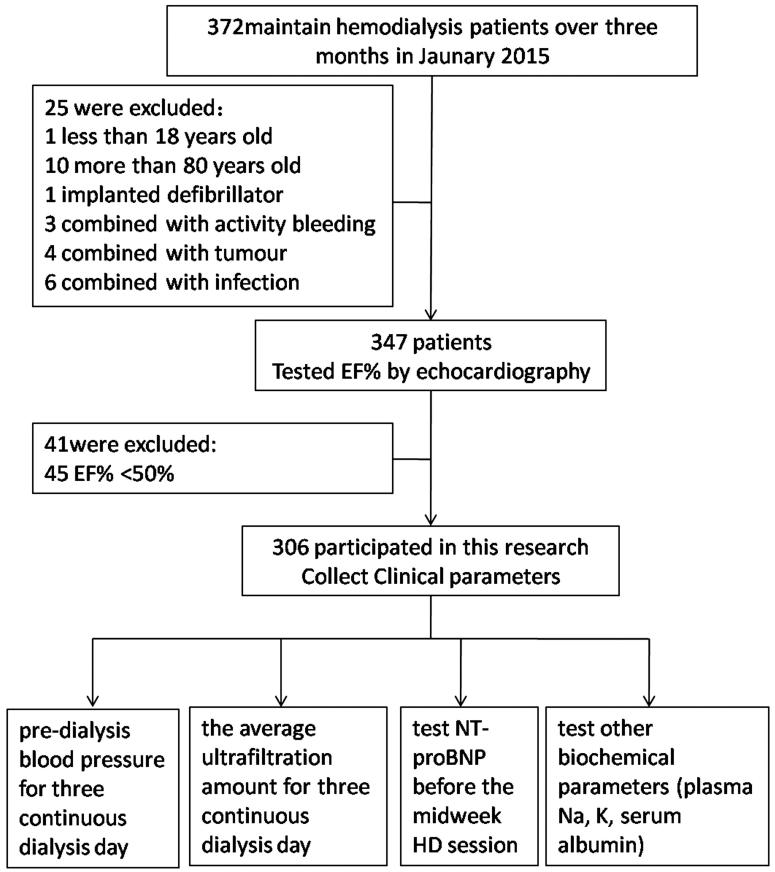
Study flowchart.

A total of 306 consecutive adult patients on HD from the HD units of Xiamen Zhongshan hospital, China were included in the study. Patients aged between 18 and 80 years with a cardiac EF ≥50% who agreed to participate in the study were included. Patients who were placed on HD <3 months before the initiation of the study; patients with EF <50%; those with a pacemaker or implanted defibrillator, amputation, and metallic prosthesis; and those with active bleeding, tumor, and infection were excluded from the study. Patients with incomplete clinical and biochemical data were also excluded. The study flowchart is shown in Figure 1.

Ethics approval was obtained from the Human Research Ethics Board at Xiamen Zhongshan Hospital. All patients included in the study provided written informed consent.

### Clinical and biochemical parameters

Clinical parameters, including pre-dialysis BP for three consecutive dialysis days, were collected. BP was measured with the patient in the supine position using the same dialysis machine, at the beginning of a dialysis session. The average ultrafiltration rate was calculated from the difference of pre-and post-dialysis weight measurement for three consecutive dialysis days. Dry weight was obtained from the patient charts. The serum samples were collected and measured before the midweek HD session and the NT-proBNP levels were centrally analyzed using the Roche Elecsys^®^ kit (Roche Diagnostics GmbH, Mannheim, Germany), an electrochemiluminescence “sandwich” immunoassay based on polyclonal antibodies against NT-proBNP. Biochemical parameters including plasma sodium, potassium, serum albumin, blood urea nitrogen, and serum creatinine were obtained from the most recent monthly blood test reports of the patients.

### Statistical analysis

Data were analyzed using the SPSS statistical software 22.0 (SPSS Inc., Chicago, IL, USA). Quantitative data were expressed as mean ± standard error of mean, and the categorical variables were expressed as absolute number and percentage. The differences between groups were compared using the Chi-squared test for discrete variables or *t*-tests for continuous variables. The categorical variables were compared using Pearson’s *χ*^2^ or Fisher’s exact test. A receiver operating characteristic (ROC) curve was applied to assess the diagnostic accuracy of NT-proBNP in the patients on HD. The area under the ROC curve (AUC) was considered a diagnostic index, and the best cutoff point was obtained based on the highest sensitivity and specificity values. *p* < 0.05 was considered statistically significant.

## Results

### General patient characteristics

A total of 306 patients (121 female, 185 male) with uremia who were maintained on regular HD were included in this study. The causes of end-stage renal disease included primary glomerulopathy (133, 43.46%), diabetic nephropathy (100, 32.68%), hypertension (35, 11.44%), lupus nephritis (4, 1.31%), autosomal dominant polycystic kidney disease (9, 2.94%), gouty nephropathy (7, 2.29%), vasculitis-related renal damage (3, 0.98%), obstructive nephropathy (2, 0.65%), chronic allograft nephropathy (3, 0.98%), tumor-associated nephropathy (1, 0.33%), hepatitis B virus-associated nephropathy (4, 1.31%), and other unknown reasons (4, 1.31%). The baseline characteristics of the patients are shown in Table 1.

**Table 1.** Characteristics of the study population (NT-proBNP, pg/mL).

Characteristics	All patients	NT-proBNP >35,000	10,000 < NT-proBNP ≤35,000	5000 < NT-proBNP ≤10,000	1000 < NT-proBNP ≤5000	500 < NT-proBNP ≤1000	NT-proBNP ≤500	*p* Values
No. (%)	306	46 (15.1)	41 (13.4)	67 (21.9)	109 (35.6)	31 (10.1)	12 (3.9)	
Age	59.14 ± 0.84	58.22 ± 2.31	59.22 ± 2.56	60.42 ± 1.79	59.52 ± 1.29	56.45 ± 2.78	58.00 ± 4.14	
Gender, female (%)	121 (39.5)	15 (32.6)	17 (41.5)	26 (38.8)	48 (44.0)	11 (37.9)	4 (44.4)	0.977
The causes of ESRD (%)								0.661
Glomerulopathy	133 (43.46)	17 (36.96)	21 (51.22)	25 (37.31)	51 (46.79)	13 (41.94)	6 (50.00)	
Diabetes mellitus	100 (32.68)	17 (36.96)	14 (34.15)	26 (38.81)	33 (30.28)	6 (19.35)	4 (33.33)	
Hypertension	35 (11.44)	7 (15.22)	5 (12.19)	9 (13.43)	10 (9.17)	4 (12.90)	1 (8.33)	
Lupus nephritis	4 (1.31)	2 (4.35)	1 (2.44)	0 (0)	0 (0)	1 (3.23)	0 (0)	
Autosomal dominant polycystic kidney disease	9 (2.94)	0 (0)	0 (0)	2 (2.98)	3 (2.75)	3 (9.68)	1 (8.33)	
Gouty nephropathy	7 (2.29)	1 (2.17)	0 (0)	1 (1.49)	4 (3.67)	1 (3.23)	0 (0)	
Vasculitis nephritis	3 (0.98)	0 (0)	0 (0)	1 (1.49)	2 (1.83)	0 (0)	0 (0)	
Obstructive nephropathy	2 (0.65)	1 (2.17)	0 (0)	0 (0)	1 (0.92)	0 (0)	0 (0)	
Chronic allograft nephropathy	3 (0.98)	0 (0)	0 (0)	0 (0)	1 (0.92)	2 (6.45)	0 (0)	
Tumor-associated nephropathy	1 (0.33)	0 (0)	0 (0)	0 (0)	1 (0.92)	0 (0)	0 (0)	
Hepatitis B virus-associated nephropathy	4 (1.31)	1 (2.17)	0	2 (2.98)	1 (0.92)	0	0	
Unknown cause	4 (1.31)	0 (0)	0 (0)	1 (1.49)	2 (1.83)	1 (3.23)	0 (0)	
Pre-HD-SBP (mmHg)	147.39 ± 1.29	150.57 ± 4.91	157.41 ± 3.58	150.21 ± 2.21	142.68 ± 2.14^b^^,^^c^	139.36 ± 3.50^b^^,^^c^	131.70 ± 6.79^a^^–^^b^^c^	<0.001
Pre-HD-DBP (mmHg)	82.35 ± 0.80	86.71 ± 3.10	83.26 ± 2.03	83.94 ± 1.51	79.59 ± 1.33	81.36 ± 2.34	72.07 ± 2.16^a^^–^^d^	<0.001
Cardiac ejection fraction (%) (EF%)	61.56 ± 0.61	61.24 ± 1.40	58.17 ± 1.70	61.48 ± 1.15	62.21 ± 1.15	61.54 ± 1.75	66.20 ± 1.93	0.136
Antihypertensive medication	205 (66.99)	39 (84.78)	34 (82.93)	53 (79.10)	63 (57.80)	13 (41.94)	3 (25.00)	<0.001
Dry weight (kg)	58.15 ± 0.68	55.19 ± 1.35	58.82 ± 1.71	57.94 ± 1.69	57.79 ± 1.12	63.65 ± 2.22^a^	58.33 ± 2.42	0.056
Ultrafiltration (L)	2.25 ± 0.05	2.57 ± 0.12	2.34 ± 0.12	2.39 ± 0.07	2.19 ± 0.08^a^	1.77 ± 0.21^a^^–^^c^	1.45 ± 0.35^a^^–^^c^	<0.001
Ultrafiltration/dry weight ratio (%)	3.95 ± 0.09	4.73 ± 0.23	4.08 ± 0.24	4.25 ± 0.14	3.86 ± 0.13	2.71 ± 0.31^a^^–^^d^	2.38 ± 0.57^a^^–^^d^	<0.001
Hemoglobin (g/L)	113.11 ± 1.13	102.74 ± 3.26	106.24 ± 3.39	115.81 ± 2.33	116.91 ± 1.64^a^^,^^b^	117.48 ± 3.02^a^^,^^b^	117.13 ± 4.77^a^	<0.001
Plasma sodium (mmol/L)	140.15 ± 0.18	141.48 ± 0.32	139.84 ± 0.73	140.91 ± 0.28	139.45 ± 0.30	139.62 ± 0.62	141.48 ± 0.96	0.118
Serum ferritin(μg/L)	409.27 ± 18.33	384.51 ± 51.25	388.22 ± 50.94	358.94 ± 42.59	440.15 ± 28.34	458.05 ± 50.71	463.15 ± 98.11	0.529
Transferring saturation (%)	36.05 ± 0.96	31.50 ± 2.14	34.48 ± 3.08	34.93 ± 2.03	38.36 ± 1.51	38.98 ± 3.37	38.58 ± 4.29	
ESA (α) dose/w kg	111.08 ± 4.86	126.84 ± 12.28	139.44 ± 10.08	107.88 ± 12.02	87.96 ± 6.41	112.74 ± 17.01	66.86 ± 23.19^a^^,^^b^	0.006
ESA (β) dose/w kg	61.21 ± 4.71	100.53 ± 9.58	73.44 ± 8.99	58.16 ± 12.92	51.51 ± 6.28	78.86 ± 7.19	31.13 ± 12.3^a^^,^^b^	0.045
Serum potassium (mEq/L)	5.12 ± 0.05	5.03 ± 0.17	5.28 ± 0.17	5.20 ± 0.10	5.14 ± 0.07	4.83 ± 0.12	4.99 ± 0.15	0.847
Albumin (g/L)	41.43 ± 0.26	39.33 ± 0.75	42.35 ± 0.53	40.64 ± 0.63	41.84 ± 0.37	43.59 ± 0.70	41.89 ± 1.08	0.056
BUN (mmol/L)	24.03 ± 0.53	23.43 ± 2.35	23.69 ± 1.11	24.16 ± 0.87	23.90 ± 0.75	24.94 ± 1.29	23.94 ± 2.84	0.484
Scr (μmol/L)	917.58 ± 17.96	831.14 ± 50.08	932.14 ± 38.04	927.15 ± 37.22	940.97 ± 28.88	992.50 ± 69.08	798.30 ± 101.48	0.065

pre-HD-SBP: pre-hemodialysis systolic blood pressure; pre-HD-DBP: pre-hemodialysis diastolic blood pressure; BUN: blood urea nitrogen; Scr: serum creatinine.

*p < 0.05.

a Significant difference from NT-proBNP >35,000 pg/mL.

b Significant difference from 10,000 pg/mL < NT-proBNP ≤35,000 pg/mL.

c Significant difference from 5000 pg/mL < NT-proBNP ≤10,000 pg/mL.

d Significant difference from 1000 pg/mL < NT-proBNP ≤5000 pg/mL.

e Significant difference from 500 pg/mL < NT-proBNP ≤1000 mL.

The patients were divided into six groups according to their pre-dialysis NT-proBNP levels; 46 patients (15.1%) had a level >35,000 pg/mL; 41 patients (13.4%) had a level between 10,000 and 35,000 pg/mL; 67 patients (21.9%) had a level between 5000 and 10,000 pg/mL; 109 patients (35.6%) had a level between 1000 and 5000 pg/mL; 31 patients (10.1%) had a level between 500 and 1000 pg/mL; and 12 patients (3.9%) had a level less than or equal to 500 pg/mL. None of the patients had a normal plasma NT-proBNP level (<100 pg/mL). There were no significant differences among the patients in the six groups with regard to age, gender, dry weight, primary diseases, and plasma levels of sodium and potassium (Table 1, *p* > 0.05).

### Relationship between NT-proBNP level and BP

There was a positive correlation between NT-proBNP level and systolic and diastolic BP (Table 1, *p* < 0.001).

The pre-dialysis systolic BP in patients with NT-proBNP >35,000 pg/mL (150.57 ± 4.91 mmHg, *n* = 46) was significantly higher than the systolic BP in patients with NT-proBNP ≤500 pg/mL (131.70 ± 6.79 mmHg, *n* = 12; *p* < 0.05). Similarly, the pre-dialysis systolic BP in patients with 10,000 pg/mL < NT-proBNP ≤35,000 pg/mL (157.41 ± 3.58 mmHg, *n* = 41) and in those with 5000 pg/mL < NT-proBNP ≤10,000 pg/mL (150.21 ± 2.21 mmHg, *n* = 67) was significantly higher than the pre-dialysis systolic BP in patients with 1000 pg/mL < NT-proBNP ≤5000 pg/mL (142.68 ± 2.14 mmHg, *n* = 109; *p* < 0.05), in those with 500 pg/mL < NT-proBNP ≤1000 pg/mL (139.36 ± 3.50 mmHg, *n* = 31; *p* < 0.05), and in those with NT-proBNP ≤500 pg/mL (131.70 ± 6.79 mmHg, *n* = 12; *p* < 0.05), as shown in Table 1.

The pre-dialysis diastolic BP in patients with NT-proBNP ≤500 pg/mL (72.07 ± 2.16 mmHg, *n* = 12) was significantly lower than that in patients with NT-proBNP >35,000 pg/mL, 10,000 pg/mL < NT-proBNP ≤35,000 pg/mL, 5,000 pg/mL < NT-proBNP ≤10,000 pg/mL, 1000 pg/mL < NT-proBNP ≤5000 pg/mL, and 500 pg/mL < NT-proBNP ≤1000 pg/mL (86.71 ± 3.10 mmHg, *n* = 46; 83.26 ± 2.03 mmHg, *n* = 41; 83.94 ± 1.51 mmHg, *n* = 67; 79.59 ± 1.33 mmHg, *n* = 109; 81.36 ± 2.34 mmHg, *n* = 31; *p* < 0.05, respectively).

Antihypertensive medication was prescribed in 68.11% of the total patients. This treatment was more commonly used in patients with NT-proBNP >35,000 pg/mL (84.78%, *n* = 46) than in those with 500 pg/mL < NT-proBNP ≤1000 pg/mL (44.83%, *n* = 31) and in those with NT-proBNP ≤500 pg/mL (33.33%, *n* = 12, *p* < 0.05).

### Relationship between NT-proBNP level and ultrafiltration/dry weight ratio

There was a positive correlation between NT-proBNP and ultrafiltration/dry weight ratio (Table 1, *p* < 0.001).

The ultrafiltration/dry weight ratio in patients with NT-proBNP >35,000 pg/mL (4.73 ± 0.23%, *n* = 46), 10,000 pg/mL < NT-proBNP ≤35,000 pg/mL (4.08 ± 0.24%, *n* = 41), and 5000 pg/mL < NT-proBNP ≤10,000 pg/mL (4.25 ± 0.14%, *n* = 67) was significantly higher than the ratio in patients with 500 pg/mL < NT-proBNP ≤1000 pg/mL (2.71 ± 0.31%, *n* = 31; *p* < 0.05) and NT-proBNP ≤500 pg/mL (2.38 ± 0.57%, *n* = 12; *p* < 0.05).

### Relationship between NT-proBNP level and cardiac ejection fraction% (EF%)

There were 81.82% patients in our HD center whose EF% over 50% included in this study. There was no significant correlation between NT-proBNP and EF% (Table 1, *p* > 0.05).

### Relationship between NT-proBNP level and hemoglobin

There was a negative correlation between NT-proBNP and hemoglobin (Table 1, *p* < 0.001).

The hemoglobin level in patients with NT-proBNP >35,000 pg/mL (102.74 ± 3.26 g/L, *n* = 46) was significantly lower than the level in patients with 1000 pg/mL < NT-proBNP ≤5000 pg/mL, 500 pg/mL < NT-proBNP ≤1000 pg/mL, and NT-proBNP ≤500 pg/mL (116.91 ± 1.64 g/L, *n* = 109; 117.48 ± 3.02 g/L, *n* = 31; 117.13 ± 4.77 g/L, *n* = 12; *p* < 0.05, respectively).

### Optimal cutoff point of NT-proBNP in patients on HD

**Figure 2.**
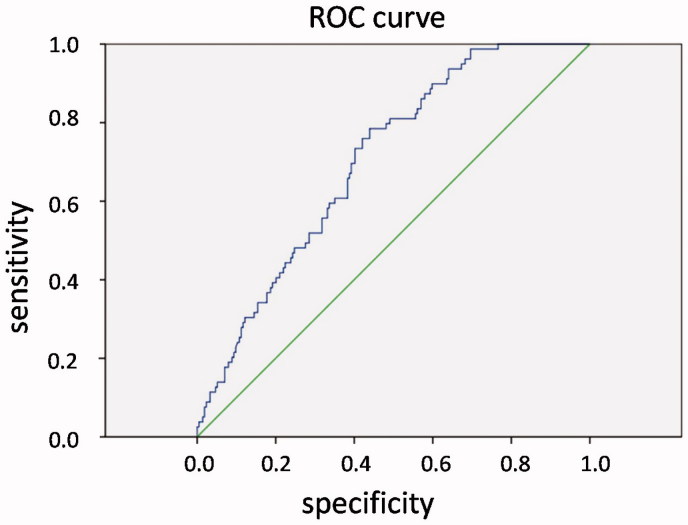
Receiver operating characteristic curve. The optimal cutoff point of NT-proBNP level for patients on maintained hemodialysis was 5666 pg/mL, with a sensitivity of 78.5%, specificity of 43.9%, and area under the curve of 0.703 (*p* < 0.001).

According to the recommendations of Kidney Disease: Improving Global Outcomes (KDIGO) and Kidney Disease Outcomes Quality Initiative (KDOQI) guidelines in patients on maintained HD, the target BP is between 90/60 mmHg and 140/90 mmHg,^25^ while the target ultrafiltration/dry weight ratio and hemoglobin are ≤4.8%,^26^ and 110–130 g/L, respectively.^27^ We observed that the optimal cutoff point of NT-proBNP level for patients on maintained HD was 5666 pg/mL, with a sensitivity of 78.5%, specificity of 43.9%, and AUC of 0.703 (*p* < 0.001, Figure 2). Thus, NT-proBNP level ≤5666 pg/mL was recommended in patients on maintained HD with EF >50%.

## Discussion

This prospective cohort study analyzed the pre-dialysis NT-proBNP levels in 306 patients on maintained HD in a single center. None of the patients had a normal plasma NT-proBNP level (<125 pg/mL). In order to investigate the prevalence of different stage NT-proBNP level and the relationship of NT-proBNP with pre-dialysis BP and ultrafiltration/dry weight ratio, as well as hemoglobin level, and to explore the optimal NT-proBNP level for predicting the best volume status in patients on HD, we included HD patients whose EF% were over 50% and divided these patients into six groups according to their NT-proBNP levels. There was no significant correlation between NT-proBNP and EF%. 15.1% patients had an extremely high NT-proBNP level (>35,000 pg/mL), 3.9% patients had an NT-proBNP level less than 500 pg/mL, while the levels of the rest of the patients were between 500 and 35,000 pg/mL. So the number of patients in each group was completely different. Thus, it seemed impossible for patients on HD to maintain an NT-proBNP level <125 pg/mL.

In this study, a correlation was observed between NT-proBNP and BP, including systolic and diastolic BP, which was consistent with a previous report.^8^ In addition, when NT-proBNP was ;<500 pg/mL, patients easily achieved the ideal pre-dialysis BP, even under the least dose of antihypertensive medication. In addition, NT-proBNP level was found to be positively associated with ultrafiltration/dry weight ratio in patients on HD, suggesting that a reduced ultrafiltration/dry weight ratio of less than 2.38 ± 0.57% facilitated achievement of the lowest NT-proBNP level (<500 pg/mL).

In this study, a reverse trend was observed between the NT-proBNP level and hemoglobin concentration. There were 69 patients treated with Epiao^®^ (epoetin-alfa, Liaoning province, China) and 237 patients treated with Recormon^®^ (epoetin-beta, Germany) in this study. In addition, we found that there were significant correlation between NT-proBNP level and erythropoiesis stimulating agents (ESA) week dose. A reverse trend was also observed between hemoglobin concentration and ESA dose (*p* < 0.001). Our findings were consistent with the findings of Ruperti et al.^28^ who found a significant reverse relationship between the NT-proBNP level with hemoglobin concentration but not volume-independent erythrocyte-related parameters, probably because of a hemodilution effect. In addition, NT-proBNP level <5000 pg/mL was recommended for patients on HD, with an aim to achieve the target hemoglobin concentration based on the KDIGO guideline.^15^

The NT-proBNP concentrations of 450 pg/mL, 900 pg/mL, and 1800 pg/mL have been reported to identify acute heart failure in patients aged <50, 50–75, and >75 years, respectively, and an NT-proBNP concentration >5180 pg/mL is strongly predictive of death by 76 days.^29^ A study performed by Ludka et al.^30^ showed that the mean absolute level of NT-proBNP in patients on HD was 2281 ± 1710 pmol/L, which was significantly higher than the normal level. In the present study, we established ROC curves to explore the optimal NT-proBNP cutoff value in patients on maintained HD, according to the recommended BP, ultrafiltration/dry weight ratio, and hemoglobin values from the KDIGO and KDOQI guidelines.^25–27^ The findings suggested that the optimal cutoff point of NT-proBNP level for patients on maintained HD with EF >50% was 5666 pg/mL. Fluid volume levels should be closely monitored in patients, especially in those with NT-proBNP level above this cutoff point.

This study had some limitations. First, this was a preliminary observational study performed in a single center, and only the NT-proBNP baseline level was observed in our patients on maintained HD. Second, the role of some unmeasured confounding factors that could have possibly influenced the observed associations, for example, pharmacological interventions such as antihypertensive medication and erythropoietin-stimulating agents, cannot be entirely ruled out. Indeed, our observations on the association between NT-proBNP and BP, hemoglobin, as well as ultrafiltration/dry weight ratio require further exploration in longitudinal prospective studies. Third, considering that this was a single-center study conducted in China, caution must be exercised when generalizing the results of this study in a different population.

In conclusion, the high NT-proBNP level was associated with high BP and ultrafiltration/dry weight ratio, and low hemoglobin level. NT-proBNP level ≤5666 pg/mL was recommended to achieve the target BP, hemoglobin level, and ultrafiltration/dry weight ratio in patients on maintained HD with an EF >50%.

## Disclosure statement

The authors have no conflicts of interest to declare.

The manuscript has been read and approved by all authors, and it is not under consideration for publication elsewhere in a similar form, in any language.
